# Epidermoid Cyst in the Submandibular Region Mimicking Plunging Ranula: A Case Report

**DOI:** 10.7759/cureus.52623

**Published:** 2024-01-20

**Authors:** Benjamin T Gillette, Cameron M Heilbronn

**Affiliations:** 1 Otolaryngology - Head and Neck Surgery, McLaren Oakland Hospital, Pontiac, USA

**Keywords:** congenital epidermoid cyst, open surgical approach, submandibular, plunging ranula, giant epidermoid cyst

## Abstract

Epidermoid cysts rarely present in the submandibular area, constituting approximately less than 7% of all cystic lesions in the head and neck region and less than 0.01% of all oral cavity cysts. Therefore, epidermoid cysts can be easily misdiagnosed, as the differential diagnosis for a submandibular area mass is very broad. Imaging can help define characteristics of the mass; however, a pathologic specimen is usually required for the final diagnosis. Surgical excision is often required and tolerated well by most patients. However, there is a risk of recurrence of the cyst after excision, as well as a rare chance for malignant transformation if not excised, which must be discussed with the patient at the time of diagnosis of epidermoid cyst. We present a 33-year-old Caucasian female with a left submandibular cystic mass measuring 4.7 cm x 2.9 cm, that was originally thought to be a plunging ranula and subsequently diagnosed as an epidermoid cyst. This report is meant to raise awareness of the possibility of a submandibular mass being an epidermoid cyst as well as appropriate workup, treatment, and prognosis of epidermoid cysts in the submandibular region. This report also describes a unique approach to a submandibular epidermoid cyst of which the submandibular gland is divided for access to the cyst for safe and effective excision. To the author's knowledge, this surgical approach has not been described in the literature for a submandibular epidermoid cyst.

## Introduction

Epidermoid cysts are rare in the head and neck, constituting approximately less than 7% of all cystic lesions in the head and neck region and less than 0.01% of all oral cavity cysts [1]. An epidermoid cyst, sometimes referred to as a sebaceous cyst, is a benign encapsulated nodule filled with keratin material often just beneath the epidermis [2]. The etiology of epidermoid cysts can essentially be broken down into two categories: congenital and acquired. Congenital cases involve ectodermal tissues that do not undergo regression whereas acquired cases typically are thought to involve the introduction of epidermal tissue into the dermis via trauma, inflammation, or surgery [3]. Epidermoid cysts appear histologically as cystic spaces lined by simple squamous epithelium. This is compared to a dermoid cyst containing both ectodermal and endodermal components or a teratoma containing all three germ cell layers [3]. Typically, these lesions will either be noticed by the patient, picked up on a physical exam, or may even be an incidental finding on imaging [1]. Imaging via computed tomography (CT) scan or magnetic resonance imaging (MRI) can both help evaluate the extent of an epidermoid cyst and surrounding anatomical structures. However, fine needle aspiration (FNA) and ultimately excisional biopsy are needed for definitive diagnosis of lesions in this area [1]. Several head and neck masses may appear similar to an epidermoid cyst; therefore, a final pathological diagnosis is important for proper treatment planning. For example, a plunging ranula may appear similar to a submandibular epidermoid cyst on both physical exam and imaging. This case report describes a submandibular cystic mass, which was initially thought to be a plunging ranula and was subsequently diagnosed as an epidermoid cyst on permanent pathology. This case presentation is particularly unique in that the surgical approach, which is thoroughly described in this report, involved dividing the submandibular gland to access the submandibular epidermoid cyst. To the author's knowledge, no other reports involving division of the submandibular gland for access and excision of a submandibular epidermoid cyst have been described in the literature.

## Case presentation

**Figure 1:**
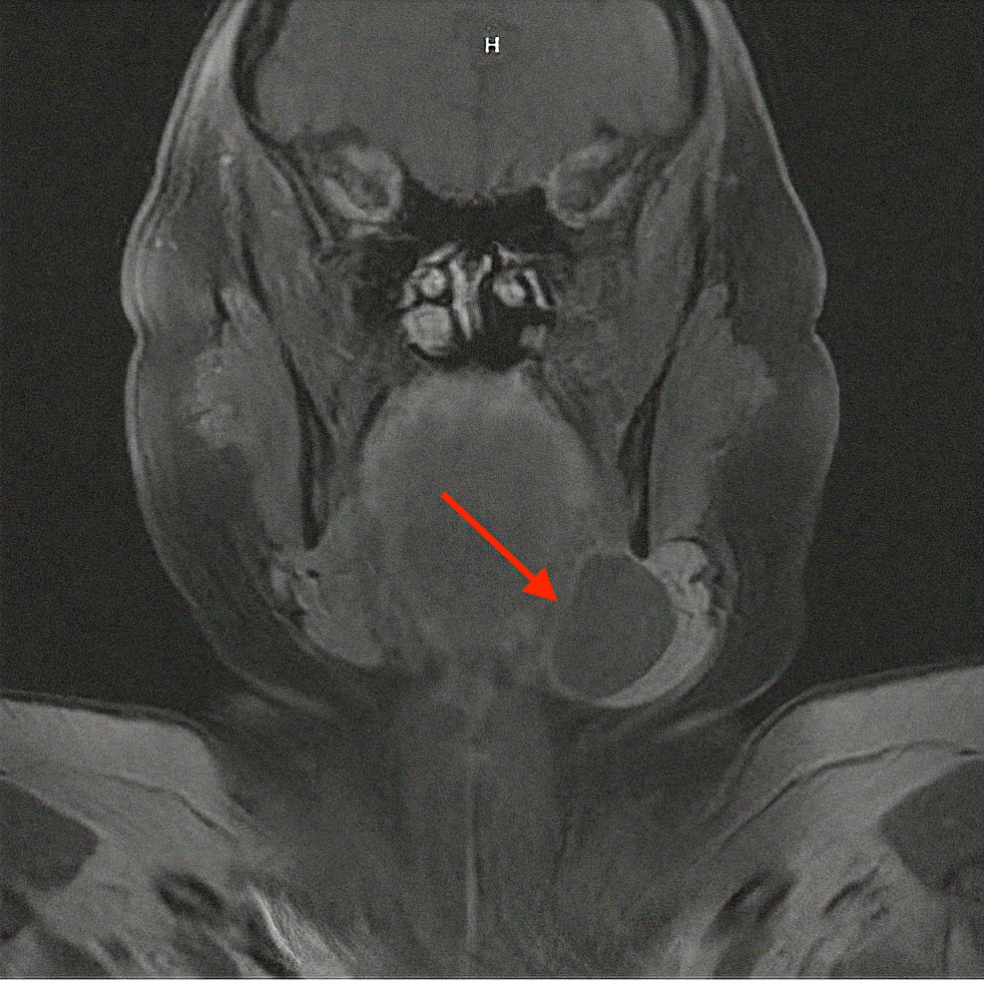
T1-weighted MRI of the head and neck with contrast, coronal cut, demonstrating a thin-walled, nonenhancing, elongated cystic mass (red arrow) measuring 4.8 cm x 2.6 cm on the medial aspect of the left submandibular gland.

**Figure 2:**
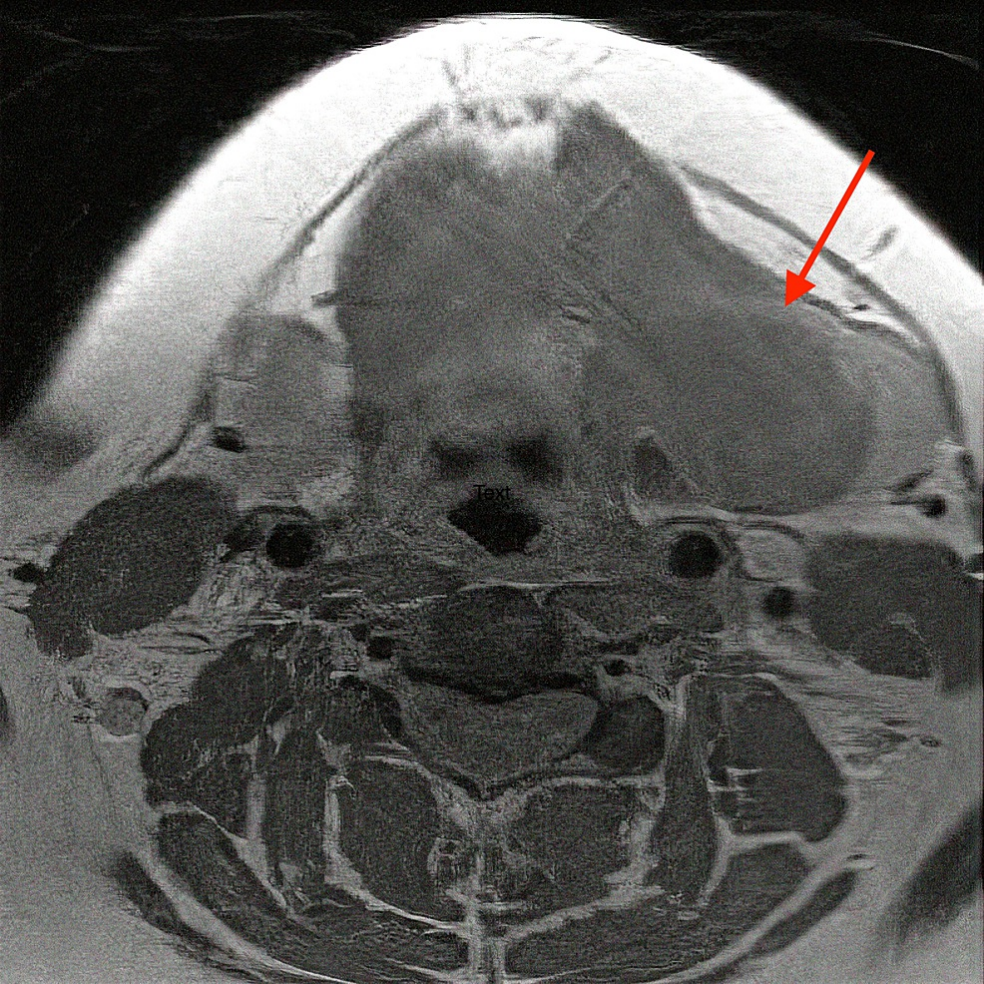
T1-weighted MRI of the head and neck with contrast, axial cut, demonstrating a thin-walled, nonenhancing, elongated cystic mass (red arrow) measuring 4.8 cm x 2.6 cm on the medial aspect of the left submandibular gland.

**Figure 3:**
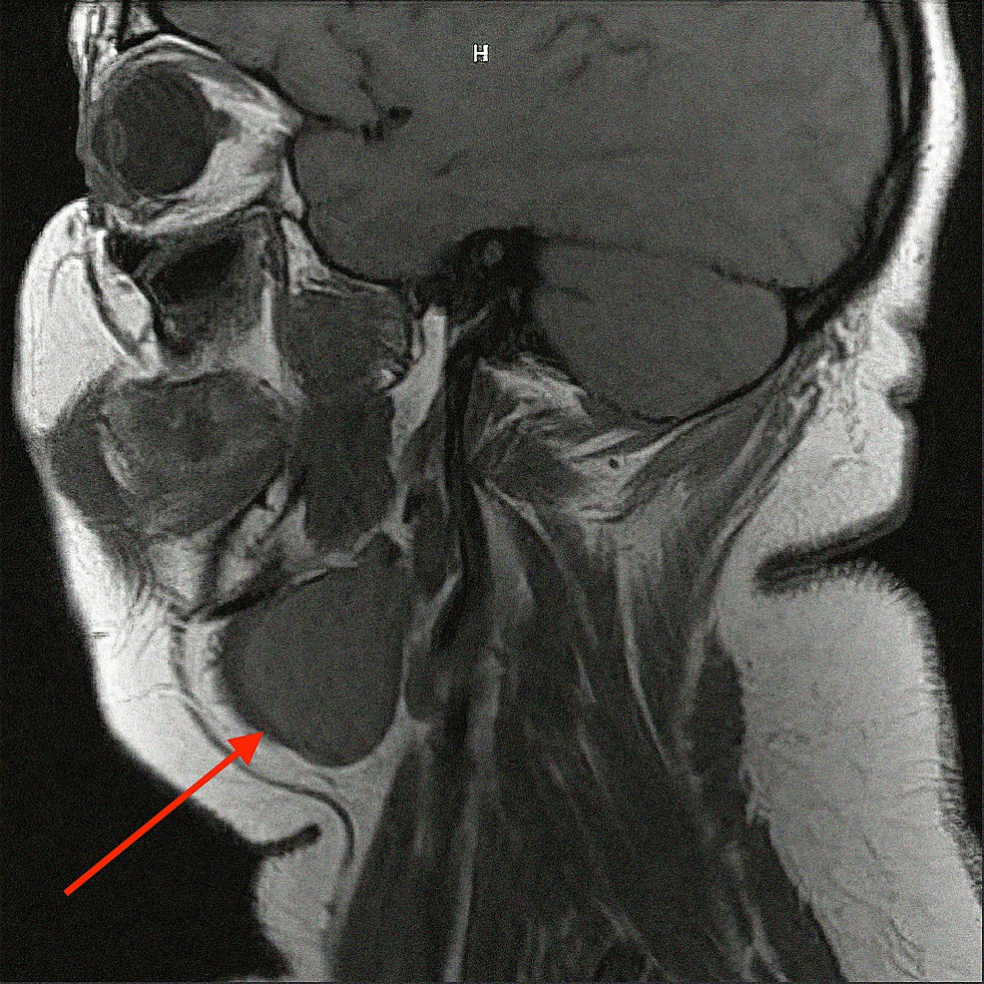
T1-weighted MRI of the head and neck with contrast, sagittal cut, demonstrating a thin-walled, nonenhancing, elongated cystic mass (red arrow) measuring 4.8 cm x 2.6 cm on the medial aspect of the left submandibular gland.

A 33-year-old Caucasian female presented for evaluation of a left submandibular mass. This submandibular mass was reportedly growing slowly over the last 1.5 years. She was not experiencing any associated symptoms such as dysphagia, dysphonia, or dyspnea, and the patient was convinced this was just fatty tissue. However, she did notice a recent increase in her snoring and left submental fullness. Before presenting to her otolaryngologist, the patient received a CT scan from her primary care provider, demonstrating a 4.7 cm x 2.9 cm left submandibular cystic mass suspicious for a plunging ranula. Physical exam at this time revealed a non-tender left submandibular ill-defined, soft, cystic mass with no associated skin changes. The patient was scheduled for left sublingual gland excision and excision of suspected plunging ranula. During intra-oral sublingual gland excision, the pocket for the suspected plunging ranula was unable to be identified intraoperatively, and ultrasound (US)-guided FNA was attempted in the post-anesthesia care unit. FNA biopsy results demonstrated almost all anucleated squamous cells, favoring epidermoid cyst content. At her two-week postoperative follow-up appointment, the patient had persistent left submental fullness. At her one-month postoperative visit, the patient had persistent left submental fullness and underwent US, which demonstrated a more solid or stippled rather than cystic appearance. MRI neck with and without contrast was then ordered and demonstrated a thin-walled, nonenhancing, elongated cystic mass measuring 4.8 x 2.6 cm on the medial aspect of the left submandibular gland (Figures 1-3).

The patient was then scheduled for definitive surgical intervention.

There are several approaches regarding the removal of masses in the neck and surgical planning aided by imaging is of utmost importance. This particular case involved making a 6 cm incision along the left neck two fingerbreadths below the mandible to avoid marginal mandibular nerve injury. The submandibular gland was identified and then the submandibular fascia was incised at the inferior aspect of the gland and elevated superiorly to protect the marginal mandibular nerve. The cystic mass was palpated deep to the submandibular gland and therefore the submandibular gland was divided in a natural plane. The facial vein was tied off with a 3-0 silk suture and divided. The anterior aspect of the mass traveled anterior and deep to the mylohyoid muscle and into the sublingual space. Blunt dissection was used to free up the mass. The hypoglossal nerve was identified deep in the mass and appeared intact. The mass was completely freed using mostly blunt dissection without violation of the floor of the mouth. The mass was sent for permanent pathology. A microfibrillar hemostatic agent was placed in the wound bed, the submandibular gland was tucked back into place to remove dead space, and the wound was closed in a layered fashion.

The final pathologic diagnosis demonstrated a 4.5 cm x 3.6 cm x 3.0 cm piece of smooth, tan-brown piece of tissue. The specimen was then inked and sectioned to reveal a smooth to wrinkled inner lining, containing brown, grumous material consistent with the epidermoid cyst. The patient was doing well at her one-week postoperative appointment with very minimal neck fullness.

## Discussion

Epidermoid cysts, also known as sebaceous cysts, are typically thought to occur on the skin as encapsulated subepidermal nodules filled with keratin [2]. The etiology of epidermoid cysts can essentially be broken down into two categories: congenital and acquired. Congenital cases involve ectodermal tissues that do not undergo regression whereas acquired cases typically are thought to involve the introduction of epidermal tissue into the dermis via trauma, inflammation, or surgery [3]. Rarely, epidermoid cysts will present midline or in the sublingual region of the floor of the mouth. Midline lesions are traditionally thought to arise congenitally because of the midline fusion of ectodermal tissues from first and second brachial arches [4]. Presentation of these cysts has also been described in the submandibular region, but they are typically located between the mylohyoid and hyoglossus muscles, suggesting the cyst originated midline floor of the mouth and then moved laterally with expansion. Occasionally, adhesions and cord-like structures can be found intraoperatively indicating movement of the cyst [4]. The differential diagnoses for a neck mass are vast and can include but are not limited to malignancy, abscess, brachial cleft cyst, or plunging ranula [5]. Imaging can help define the mass and further narrow down the etiology, but often biopsy is required for an accurate diagnosis and guide for treatment options for the mass. This patient’s initial CT findings and the location of her mass were both consistent with the diagnosis of plunging ranula, but final pathology was required for a definitive diagnosis.

A plunging ranula typically appears as a translucent bluish swelling under the tongue and can present as a neck mass in the submandibular area. Classically, a plunging ranula appears as an unilocular water density with a smooth capsule, no septations, and may have a “tail sign” between the sublingual and submandibular spaces on CT, US, and MRI [5]. FNA will typically yield mucus high in amylase [5]. As described, upon initial clinical presentation, it is easy to misdiagnose an epidermoid cyst in the submandibular area as a plunging ranula.

Regarding the prognosis of epidermoid cysts, it is important to understand that surgical excision is required, but often these cysts can recur. There is also a minor possibility of malignant transformation with epidermoid cysts, if not excised. Both risks should be explained to the patient upon diagnosis. Epidermoid cysts in the submandibular area ultimately require surgical removal and surgical approaches vary. The literature does not often discuss surgical approaches to these cases and here we provide a unique approach that adds to the growing body of literature on the subject.

## Conclusions

Epidermoid cysts rarely present as a submandibular mass and therefore are at risk for misdiagnosis and treatment. This case not only provides awareness of this entity in the differential diagnosis of a submandibular mass but also provides a novel approach to safely excising a submandibular epidermoid cyst by dividing the submandibular gland via a transcervical approach for improved access to the cyst.
